# Community‐Based Health Insurance and Universal Health Coverage in Africa: A Commentary

**DOI:** 10.1002/puh2.70234

**Published:** 2026-04-10

**Authors:** Abraham Fessehaye Sium, Malede Birara, Meseret Olana Jeldu, Don Eliseo Lucero‐Prisno

**Affiliations:** ^1^ Department of Obstetrics and Gynecology St Paul's Hospital Millennium Medical College Addis Ababa Ethiopia; ^2^ Department of Global Health and Development, Faculty of Public Health and Policy London School of Hygiene and Tropical Medicine London UK; ^3^ Office for Research, Extension and Innovations Bukidnon State University Malaybalay City Bukidnon Philippines; ^4^ Center for Research and Development Cebu Normal University Cebu City Philippines

**Keywords:** Africa, community‐based health insurance, low‐ and middle‐income countries (LMICs), SDGs, universal health coverage

## Abstract

Community‐based health insurance (CBHI) has been shown to improve access to essential health services, including maternal and child care, in low‐ and middle‐income countries. However, its implementation across Africa remains limited. This commentary examines the role of CBHI in advancing universal health coverage in Africa, identifies barriers to its wider adoption, and proposes strategies to overcome them.

## 1 Introduction

Economic development is closely linked to population health. African countries have tried different modes of health‐care financing since independence [1]. Community‐based health insurance (CBHI) has evolved as an appropriate risk‐pooling mechanism to populations without access to formal health insurance in low‐middle‐income countries, including in Africa [2, 3]. Evidence also shows that this essential health‐care financing strategy contributes to improved access to essential health care in the continent of Africa [4, 5].

CBHI schemes in Rwanda are one of the largest experiments in community‐based risk‐sharing mechanisms in sub‐Saharan Africa for health‐related problems. Evidence shows that the utilization of health‐care services is high among insured households in Rwanda. CBHI also significantly decreases the amount of per capita spending on health care in the country [6, 7]. Another example is the case of Ethiopia. A systematic review and meta‐analysis reveals that CBHI scheme significantly increases universal health coverage, whereas it decreases catastrophic expenditure by more than 70% [8]. Ethiopia introduced CBHI in 2011 [9]. Similarly, a large survey study from three West African countries (Senegal, Mali, and Ghana) demonstrates the benefits of CBHI—membership in a CBHI scheme is positively associated with the use of maternal health services [10]. This evidence is substantiated by findings of another recent systematic review that reveals CBHI could serve as a long‐term strategy to alleviate West Africa's burden of high maternal and child mortality rates [11].

Despite its game‐changer role in expanding universal health coverage as well as protecting against catastrophic health‐care expenditure, the utilization of CBHI across the continent of Africa remains low. There are multiple barriers at individual level (lack of awareness, beliefs, and perceived health status), community level (lack of trust and socioeconomic factors), and system level (poor service quality and limited health benefit entitlements) [1, 12]. There is a need for synergistic action from multiple actors to mitigate these barriers.

## 2 Conclusion

Scaling up CBHI is essential to achieving universal health coverage in Africa. With only 5 years remaining to meet the 2030 Sustainable Development Goals, urgent action is required to strengthen health‐financing mechanisms and ensure equitable access to care. We call on multi‐sector collaboration or policy reforms.

## Author Contributions

Abraham Fessehaye Sium and DonEliseoLucero‐Prisno III developed the concept and design of the article. Abraham Fessehaye Sium and DonEliseoLucero‐Prisno III contributed to the original manuscript development. Malede Birara and Meseret Olana Jeldu contributed to the manuscript review and writing. The final manuscript was edited by Abraham Fessehaye Sium and DonEliseoLucero‐Prisno III.

## Funding

The authors have nothing to report.

## Ethics Statement

The authors have nothing to report.

## Consent

The authors have nothing to report.

## Conflicts of Interest

The authors declare no conflicts of interest.

## Data Availability

All data supporting the conclusions of this article are included within the manuscript.
